# Functional Characterization of the *Plasmodium falciparum* Chloroquine-Resistance Transporter (*Pf*CRT) in Transformed *Dictyostelium discoideum* Vesicles

**DOI:** 10.1371/journal.pone.0039569

**Published:** 2012-06-19

**Authors:** Janni Papakrivos, Juliana M. Sá, Thomas E. Wellems

**Affiliations:** Laboratory of Malaria and Vector Research, National Institute of Allergy and Infectious Diseases, National Institutes of Health, Bethesda, Maryland, United States of America; Pennsylvania State University College of Medicine, United States of America

## Abstract

**Background:**

Chloroquine (CQ)-resistant *Plasmodium falciparum* malaria has been a global health catastrophe, yet much about the CQ resistance (CQR) mechanism remains unclear. Hallmarks of the CQR phenotype include reduced accumulation of protonated CQ as a weak base in the digestive vacuole of the erythrocyte-stage parasite, and chemosensitization of CQ-resistant (but not CQ-sensitive) *P. falciparum* by agents such as verapamil. Mutations in the *P. falciparum* CQR transporter (*Pf*CRT) confer CQR; particularly important among these mutations is the charge-loss substitution K→T at position 76. *Dictyostelium discoideum* transformed with mutant *Pf*CRT expresses key features of CQR including reduced drug accumulation and verapamil chemosensitization.

**Methodology and Findings:**

We describe the isolation and characterization of *Pf*CRT-transformed, hematin-free vesicles from *D. discoideum* cells. These vesicles permit assessments of drug accumulation, pH, and membrane potential that are difficult or impossible with hematin-containing digestive vacuoles from *P. falciparum-*infected erythrocytes. Mutant *Pf*CRT-transformed *D. discoideum* vesicles show features of the CQR phenotype, and manipulations of vesicle membrane potential by agents including ionophores produce large changes of CQ accumulation that are dissociated from vesicular pH. *Pf*CRT in its native or mutant form blunts the ability of valinomycin to reduce CQ accumulation in transformed vesicles and decreases the ability of K^+^ to reverse membrane potential hyperpolarization caused by valinomycin treatment.

**Conclusion:**

Isolated vesicles from mutant-*Pf*CRT-transformed *D. discoideum* exhibit features of the CQR phenotype, consistent with evidence that the drug resistance mechanism operates at the *P. falciparum* digestive vacuole membrane in malaria. Membrane potential apart from pH has a major effect on the *Pf*CRT-mediated CQR phenotype of *D. discoideum* vesicles. These results support a model of *Pf*CRT as an electrochemical potential-driven transporter in the drug/metabolite superfamily that (appropriately mutated) acts as a saturable simple carrier for the facilitated diffusion of protonated CQ.

## Introduction

The advent and spread of CQ-resistant *Plasmodium falciparum* malaria marked a great global health catastrophe of the 20^th^ century. Yet, even as effective replacements for CQ are sought in pharmaceutical research, much about the CQR mechanism remains unclear. Hallmarks of the CQR phenotype include reduced CQ accumulation in the digestive vacuole (DV) of the parasite [1], [2] and chemosensitization of CQ-resistant but not CQ-sensitive parasites by such agents as verapamil (VP), tricyclic antidepressants, phenothiazines, antihistamines, primaquine and various plant alkaloids [3]–[8]. At the genetic level, mutations in the parasite-encoded *P. falciparum* CQR transporter (*Pf*CRT) confer CQR; particularly important among these mutations in naturally resistant parasites is the charge-loss substitution K→T at position 76 [9], [10]. *Pf*CRT may be phosphorylated for trafficking [11], spans the membrane of the DV with ten predicted transmembrane domains [9] and is a member of the drug metabolite/transporter (DMT) superfamily of electrochemical potential-driven transporters [12]–[14] (http://www.tcdb.org/browse.php). Evidence for the importance of a charge change in substrate recognition by *Pf*CRT has been reinforced by the findings of point mutations K76N and K76I in CQ-resistant parasites selected from the CQ-sensitive Sudan line 106/1 [15]. Conversely, CQ-sensitive parasites with novel positively-charged *Pf*CRT mutations have been obtained from CQ-resistant parasites by stepwise selection with the antiviral agent amantadine or the antimalarial agent, quinine (QN) [16], [17].

CQ is a lipophilic weak base with two basic ionization sites reported to have pK_a_ values of 10.4 and 8.1, respectively, at 37°C [18]. Although the concentration of the unprotonated form of CQ is calculated to be less than 0.01% at neutral pH, it has generally been believed that the drug diffuses by this neutral form across lipid bilayers of the cell into acidic compartments such as the DV, where it can become trapped due to its greater protonation at lower pH (Henderson-Hasselbalch equilibrium) [19]–[21]. Yet CQ is also reported to reach millimolar concentrations in the parasite DV, a level difficult to explain by weak base partitioning alone [22]. An additional component of CQ accumulation has been associated with the digestion of hemoglobin and release of toxic ferriprotoporphyrin IX (FP IX) in the DV, where this otherwise toxic compound is converted to hematin and detoxified by crystallization into hemozoin [23]. Strong binding of CQ to hematin interferes with hemozoin formation and contributes to the high drug concentrations found in the DV of sensitive parasites after CQ treatment [19], [22].

CQ efflux from the DV of resistant parasites is thought to occur by facilitated diffusion of protonated CQ down its electrochemical gradient [24], [25]. The mutated *Pf*CRT has been proposed to promote this efflux either as a voltage gated channel [26], [27] or as a simple carrier [28]–[30] that may have been converted from an exchange-only to a net transporting function [31]. More recent reports of temperature-dependent, saturable drug transport by mutant *Pf*CRT at clinically relevant CQ concentrations [25] and of specific effects of different quinoline compounds, peptides and amantadine on *Pf*CRT function [30], [32] have weighed in favor of a carrier model. Carrier saturation may explain observations in Guinea-Bissau that CQR can be overcome by high level CQ administration in twice daily doses [33], and carrier-mediated facilitated diffusion of protonated drug is consistent with evidence that *Pf*CRT mutations can give rise to a CQ-associated “H+ leak” from the *P. falciparum* DV [14], [34], [35].

Reports from several studies have concluded that the CQR phenotype depends upon a transmembrane potential that is normally positive in its gradient from the inside of the DV (Δψ) [26], [27], [30], [36], [37]. Consistent with these conclusions, use of the mitochondrial protonophore FCCP or glucose starvation to deplete this gradient produced comparable CQ accumulation in CQ-resistant and CQ-sensitive parasites [26]. While some of these findings have been taken to support a PfCRT channel, the carrier hypothesis of facilitated diffusion remains an alternative and more likely model in view of bioinformatic analyses and the saturation and temperature-dependent kinetics noted above [24], [38].

The capacity of hematin to bind CQ in the *P. falciparum* DV and the fact that DVs are difficult to isolate in quantity from parasitized erythrocytes have complicated biochemical characterizations of *Pf*CRT. A number of research groups have therefore obtained functional information on wild-type and different mutant forms of *Pf*CRT expressed in heterologous systems including *Xenopus laevis* oocytes, *Dictyostelium discoideum*, and *Pichia pastoris* and *Saccharomyces cerevisiae* yeast [25], [27], [30], [36], [39]–[41]. *Pf*CRT-transformed *D. discoideum* cells, which contain large acidic vesicles and can be readily manipulated under experimental conditions, were shown to have features of the CQR phenotype including reduced drug accumulation and VP reversal [41]. Here we describe further characterization of the CQR phenotype in transformed *D. discoideum* and report the isolation and characterization of acidic vesicles from these cells. Our results show that CQ accumulation in the acidic vesicles of *D. discoideum* responds to membrane potential as well as pH changes. We discuss the implications of these findings in the context of present understanding of *Pf*CRT and its function in CQ transport.

## Materials and Methods

### *D. discoideum* Lines, Culture and Quality Controls

Axenic *D. discoideum* cells (AX2) were cultivated at 20°C in D3-T basic media (KD Medical, Columbia, MD, USA) and harvested for experiments during exponential growth phase (2–3×10^6^ cells/ml). Cells were transformed with plasmids pEXP4 to express full-length *Pf*CRT as described previously [41]. Banks of cryopreserved master and working lines were established to assure the quality and functionality of all cells used for experiments. *Pf*CRT expression and presence of the appropriate CQR phenotype were confirmed by PCR detection, immunoblotting and drug uptake assays for each master *D. discoideum* line, multiple samples of which were cryopreserved in liquid nitrogen. Individual samples from the master bank of *D. discoideum* lines were expanded periodically, confirmed by immunoblotting and drug uptake assays, and stored in working banks at −80°C. Cells from these −80°C banks were thawed as needed and placed into culture to generate the required populations for experiments. Before vesicle purification, drug uptake assays were performed to confirm presence of the appropriate CQR phenotype in each of these populations [41].

### Isolation and Storage of *Pf*CRT-enriched Acidic Vesicles

Exponential growth-phase cells were collected by centrifugation at 500×*g* for 5 min at 4°C and washed with D3-T basic media. Packed cells were resuspended in 5 vol vesicle preparation buffer (20 mM HEPES/Tris pH 7.3, 1 mM dithiothreitol, 1 mM MgCl_2_, 6 mM K_2_SO_4_, 6 mM NaCl, and 210 mM sucrose) and held on ice for 5 min. The suspension was passed twice through a 25-mm 5.0 μm polycarbonate filter membrane (Sigma-Aldrich, St. Louis, MO, USA) and immediately centrifuged at 1000×*g* for 5 min at 4°C to clear nuclei and unbroken cells. Pellets of the cellular material were collected from the supernatant in four steps of differential speed centrifugation at 4°C (2000×*g*, 4000×*g*, and 20,000×*g* each for 10 min, 100,000×*g* for 1 h). Protein concentrations of the resuspended pellets and the final supernatant were determined using a standard Bradford assay (Bio-Rad, Hercules, CA, USA) to ensure quantitative loading of each well prior to standard SDS-PAGE and immunoblotting. Amounts containing 15 µg of total protein were loaded for each fraction. *Pf*CRT was detected using polyclonal anti-*Pf*CRT-rabbit antiserum [9]. Fixation and preparation of samples for transmission electron microscopy observation were performed by standard procedures [9]. For vesicle preservation and storage, the pellets were snap-frozen on dry ice and stored at –80°C. The quality and function of vesicles from all preparations were routinely confirmed by CQ uptake and protein quantification assays. At least three batches of vesicles were prepared from each cell line to determine average values and standard deviations in each experiment.

### pH Measurements of Intact Cells and Acidic Vesicles

Exponential growth-phase cells were pelleted (500×*g*, 5 min) and washed twice in 10 mM phosphate buffer pH 7.5 (PB). Cells (1.1×10^8^/ml) were then incubated for 1 h in either PB or PB supplemented with 80 μM VP, 2 μM carbonylcyanide-m-chlorophenylhydrazone (CCCP), or 100 nM concanamycin A (CMA) (all chemicals from Sigma-Aldrich). Cells (1.1×10^7^) were transferred to a 2.5-ml disposable cuvette (Sigma-Aldrich), which was inserted into the magnetic-stirring mount of a fluorimeter (Photon Technology International [PTI], Birmingham, NJ, USA). Photomultiplier settings were 1× Gain μA/Volts, 0.05 time constant at 1100 Volts. Cells were kept in suspension by magnetic stirring. Estimates of vesicular pH within cells were obtained from fluorescence signals after the addition of 0.5 μM LysoSensor DND-167 (Molecular Probes, Eugene, OR, USA), a weak base with pH-dependent excitation and emission peaks of 375 and 425 nm, respectively. Data were collected and analyzed using Felix32 software (PTI).

Vesicles were obtained from exponential growth-phase cells incubated for 2 h in D3-T basic media containing 2 mg/ml fluorescein isothiocyanate (FITC)-dextran (Sigma-Aldrich). The cells were washed twice in D3-T media, pelleted, and processed as described above for the isolation of *Pf*CRT-enriched acidic vesicles. Vesicles equivalent to 300 μg total protein were suspended in 2 ml of desired buffer (indicated in Results) and incubated at 22°C with continuous monitoring for fluorescence curves or until measurement of final fluorescence end point values after 10 min. Ratios of emitted fluorescence at 518 nm after exposure to 450 and 494 nm excitation were obtained and compared with known ratios from dye calibration curves of the fluorescence of 1 mg/ml FITC in buffers of different pH (100 mM sodium acetate/acetic acid pH 4 to 5.6; 100 mM dibasic/monobasic sodium phosphate and pH 6 to 7.6) [42], [43]. Although the accuracy of pH determinations from these ratios might be altered by intravesicular conditions that are different from those of the calibration buffers, the relative pH determinations from the vesicles proved to be highly reproducible and they provided the consistency necessary for the comparative purposes of the experiments reported here.

### [^3^H]-Drug Measurements in *D. discoideum* Whole Cells and Acidic Vesicles

[^3^H]-CQ, [^3^H]-QN and [^3^H]-piperaquine (PPQ) were from American Radiolabeled Chemicals, Inc. (St. Louis, MO). Drug uptake assays with intact cells were performed in PB (10 mM Na_2_HPO_4_, 10 mM KH_2_PO_4_, pH 7.5) as previously described [41] using 100 nM [^3^H]-drug and, where indicated, 80 μM VP, 2 μM CCCP, or 100 nM CMA. Drug uptake assays with isolated *Pf*CRT-enriched acidic vesicles were performed using vesicles equivalent to 75 μg total protein. Stored frozen vesicles were thawed at room temperature (RT) for 1 min and transferred to ice for 4 min. For each drug uptake experiment, vesicles were suspended in 250 μl vesicle suspension buffer (VSB; 20 mM HEPES/Tris pH 7.3, 1 mM dithiothreitol, 1 mM MgCl_2_, 1 mM ATP, 6 mM K_2_SO_4_, 6 mM NaCl, 210 mM sucrose and 10 mg/ml bovine serum albumin for surface tension reduction). For some experiments, VSB without ATP was used as indicated, and for others the 6 mM K_2_SO_4_ and 6 mM NaCl of VSB were replaced with 30 mM sucrose (minimum cation buffer; MCB; total sucrose concentration 240 mM). In experiments designed for osmotic shock, vesicles were suspended as indicated for 5 min in a modified form of VSB containing only 100 mM sucrose (osmotic shock buffer, OS). Isoosmolarity was then reestablished by adding an equal volume of VSB containing 320 mM sucrose for an additional 5 min. In experiments with valinomycin, nigericin, or monensin (Sigma-Aldrich), vesicles in VSB were incubated with 2 μM concentrations of these compounds for 10 min at 22°C and quickly transferred onto ice before centrifugation at 100,000×*g* for 2 min at 4°C (Optima TLX ultracentrifuge; Beckman-Coulter, Fullerton, CA, USA). After removal of the supernatant by aspiration, the pellets were solubilized by suspension in 50 μl of NCS-II tissue solubilizer (Amersham, Arlington Heights, IL, USA) for 2 h or overnight at RT. The solubilized samples were snap-frozen in their tubes on dry ice, and the bottoms of the tubes containing samples were cut into 4-ml scintillation vials (PerkinElmer, Foster City, CA, USA). Ten μl of acetic acid and 3 ml of LSC-cocktail (Ultima Gold AB; PerkinElmer) were added, and [^3^H] scintillation counts were obtained in a Wallac Trilux 1650 Beta scintillation counter (Perkin Elmer).

### Determination of Cytoplasmic ATP Levels

*D. discoideum* cells (1.1×10^8^ cells/ml) were incubated in PB or PB supplemented with 80 μM VP, 2 μM CCCP, or 100 nM CMA for 1 h under standard culture conditions. ATP levels were detected using a bioluminescent assay kit (Sigma-Aldrich). Briefly, 2×10^8^ cells were pelleted and processed as described for the preparation of *Pf*CRT-enriched vesicles. Centrifugation at 100,000×*g* for 1 h yielded supernatants containing membrane-free cytoplasm. Aliquots of 100 μl were snap-frozen on dry ice and stored at –80°C. For ATP analysis, supernatants containing cytoplasm were rapidly thawed in a water bath at RT, transferred to ice, and diluted 1000-fold in VSB. Dilute supernatants (100 μl) were mixed with 100 μl 25-fold diluted ATP assay mix, incubated at RT for 3 min, and then measured in a standard luminometer (TD 20/20; Turner Design, Sunnyvale, CA, USA).

### Membrane Potential Assessments

Vesicles equivalent to 150 μg protein were suspended in 2 ml MCB buffer and transferred into a 2.5-ml disposable cuvette, which was placed into the mount of the PTI fluorimeter. Photomultiplier settings were as described for pH measurements. Vesicles were kept in suspension by magnetic stirring for 50 s before DiOC_5_(3) (Molecular Probes; 1 mM stock in 20 μl DMSO) was added to a final concentration of 250 nM. Fluorescence changes indicative of changes in membrane potential were monitored at emission wavelength 509 nm following excitation of 484 nm. Potassium chloride, ATP, or weak bases were added at time points and concentrations as indicated in the text. Data analysis was performed using Felix32 software (PTI).

## Results

### Effects of Weak Bases on the CQR Phenotype of Transformed *D. discoideum* Cells

**Figure 1:**
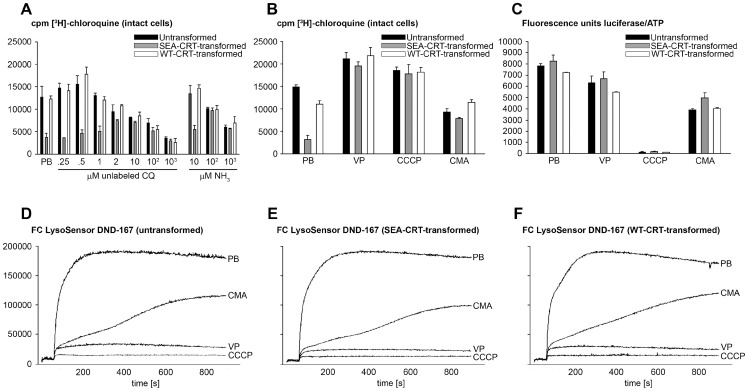
Measures of [^3^H]-CQ uptake, ATP levels, and vesicle pH in untransformed, SEA-CRT-, and WT-CRT-transformed *Dictyostelium discoideum* whole cells. (**A**) Labeled drug uptake by *D. discoideum* cells in PB containing 100 nM [^3^H]-CQ and various concentrations of unlabeled CQ and ammonia. Cells were incubated in [^3^H]-CQ for 10 min before determination of [^3^H]-CQ uptake. (**B**) Effects of VP, the protonophore CCCP, and the V-Type ATPase inhibitor CMA on [^3^H]-CQ uptake. (**C**) Effects of VP, CCCP, and CMA on cytoplasmic ATP levels. (**D–F**) Relative effects of VP, CCCP, and CMA on acidic compartment pH traced by 0.5 µM LysoSensor^TM^ Blue DND-167 probe (FC, fluorescence counts; y-axis scales are the same for figures D–F). Slight decreases of fluorescence from cells in PB after 600 s may be due to lysosomal alkalinization from the LysoSensor^TM^ Blue DND-167 probe. Concentrations of 80 μM VP, 2 μM CCCP, and 100 nM CMA were employed in the experiments. Error bars indicate standard deviations from three independent measurements.

Previous trans stimulation studies showed that pre-equilibrating *P. falciparum*-parasitized erythrocytes with unlabeled CQ in the μM range could partially reverse the phenotype of reduced [^3^H]-CQ accumulation in CQ-resistant *P. falciparum* [28]. To test for a comparable effect of pre-established vacuolar concentrations of unlabeled CQ on the features of CQR transferred to *D. discoideum*, we performed similar [^3^H]-CQ accumulation experiments with untransformed *D. discoideum* cells, cells transformed with wild-type *Pf*CRT (WT-CRT; from CQ-sensitive parasite HB3), and cells transformed with a mutated form of *Pf*CRT (SEA-CRT; from CQ-resistant parasite Dd2) [41]. After these cells were pre-loaded for 5 min with different concentrations of unlabeled CQ and then exposed to 100 nM [^3^H]-CQ for 10 min, accumulation of the [^3^H] labeled drug was determined as a measure of competition between the CQ species for transport by *Pf*CRT. Levels of [^3^H]-CQ accumulation showed a clear dependence on the concentration of unlabeled CQ (Figure 1A). In the absence of unlabeled CQ (PB control), [^3^H]-CQ accumulation was reduced ∼70% in SEA-CRT-transformed relative to untransformed *D. discoideum*, and little or no difference of accumulation was observed between WT-CRT-transformed and untransformed *D. discoideum*. SEA-CRT-transformed cells showed increasing accumulations of [^3^H]-CQ in the presence of unlabeled drug concentrations up to about 10 µM, where [^3^H]-CQ accumulation in SEA-CRT-transformed cells approached the accumulations seen in untransformed and WT-CRT-transformed cells (Figure 1A). We also noted that [^3^H]-CQ accumulations in untransformed cells and in cells transformed with WT-CRT were slightly increased in the presence of 0.25 and 0.5 µM CQ, but these decreased at concentrations above 1 µM. At unlabeled CQ concentrations of 10 µM, 100 µM, and 1000 µM, the accumulations of [^3^H]-CQ in SEA–CRT-transformed, WT–CRT-transformed, and untransformed cells were comparable to each other but, at these different concentrations, averaged only ∼66%, 50%, and 30% of the accumulation of untransformed cells in PBS alone (Figure 1A). Although the higher (10–1000 µM) concentrations of unlabeled CQ eliminated the accumulation differences between the SEA-CRT-transformed, WT-CRT-transformed, and untransformed cells, these concentrations of unlabeled CQ did not show the ability of VP to increase [^3^H]-CQ accumulation in all cell lines (Figure 1B) [41].

We also examined the effects of different concentrations of ammonia on [^3^H]-CQ accumulation in SEA-CRT-transformed relative to WT-CRT-transformed and untransformed *D. discoideum*. The weak base action of ammonia has been shown to effectively neutralize the pH of acidic vesicles in *D. discoideum* [44]. While 10 µM ammonia had little effect on [^3^H]-CQ accumulation in our three *D. discoideum* lines, 100 µM ammonia eliminated accumulation differences among these lines by increasing [^3^H]-CQ accumulation in SEA-CRT-transformed cells and decreasing [^3^H]-CQ accumulation in WT-CRT-transformed and untransformed cells (Figure 1A). Exposure to 1000 µM ammonia further decreased [^3^H]-CQ accumulation in all three lines. These results are consistent with a large effect of vesicular pH on SEA-CRT-mediated CQ accumulation by the untransformed and transformed *D. discoideum* cells.

The ability of CQ-resistant *P. falciparum* to reduce intracellular CQ levels is sensitive to treatments that affect pH and proton electrochemical gradients across the DV membrane. Treatment with protonophores, for example, can raise CQ accumulation in CQ-resistant–and lower it in CQ-sensitive–parasites [26]. We examined the influence of agents that collapse proton gradients on the CQ accumulation of SEA-CRT-transformed, WT-CRT-transformed, and untransformed *D. discoideum* cells. Relative to no agent in PB control buffer, VP and the protonophore CCCP each increased CQ accumulation in all cells and proved similar in their ability to equalize CQ accumulation in SEA-CRT-transformed relative to untransformed and WT-CRT-transformed cells (Figure 1B). The V-type ATPase inhibitor CMA likewise reversed the reduced accumulation phenotype of SEA-CRT transformed *D. discoideum*, although without the overall increased accumulation levels observed with VP and CCCP.

### Effects of CCCP, CMA, and VP on *D. discoideum* ATP Levels

CCCP inhibits mitochondrial ATP production and depletes V-type and other ATPases of substrate [45]. CMA is thought to act more specifically as an inhibitor of V-type ATPases [46]. The actions of VP are much less well understood, although we have found that VP actions include neutralization of vesicle acidity in *D. discoideum* (results below). To further explore the effects of these agents, we determined the cytoplasmic ATP levels of our untransformed and transformed *D. discoideum* lines after treatment with CCCP, CMA, or VP. In agreement with the reported effects of CCCP, we found that ATP was fully depleted from cells exposed to this agent (Figure 1C). Cells treated with CMA contained 40% less cytoplasmic ATP, and VP-treated (80 µM) cells contained 20% less ATP. With each of these agents, our results showed little or no difference between the ATP levels of untransformed, WT-CRT-transformed, or SEA-CRT-transformed cells.

### Effects of CCCP, CMA, and VP on the pH of Vesicles in *D. discoideum* Cells

The effects of CCCP, CMA, and VP on [^3^H]-CQ accumulation led us to also examine the effects of these agents on vesicular pH in untransformed, WT-CRT-transformed, and SEA-transformed *D. discoideum* cells. For this purpose, we used LysoSensor^TM^ Blue DND-167, a commercially available weak base that exhibits strong fluorescence in acidic compartments [47]. In control experiments with the untransformed cells, addition of the LysoSensor^TM^ probe produced a steep increase of fluorescence over a period of 3 min, indicative of rapid dye uptake by acidic vesicles (Figure 1D). CMA dramatically slowed this increase of fluorescence, which gradually approached a plateau after 15 min. This finding is consistent with blocked *D. discoideum* V-type ATPase activity, compromised vesicle acidification, and slowed dye uptake in the presence of CMA [48]. Little or no increase of LysoSensor^TM^ fluorescence was detected in cells exposed to VP or CCCP, consistent with marked loss of vesicular acidity with these agents (Figure 1D). Figure 1, E and F, shows results from LysoSensor^TM^ Blue DND-167 uptake experiments with SEA-CRT-transformed or WT-CRT-transformed cells. The tracings from both of these transformed lines were similar to those of untransformed cells.

The evidence for loss of vesicular acidity in VP- or CCCP-treated cells, which nevertheless can accumulate greater amounts of CQ than cells treated with CMA or control cells in PB (Figure 1B), indicates that a factor other than vesicular pH has a key role in CQ accumulation by *D. discoideum* cells.

### Isolation of Acidic Vesicles from Untransformed and *Pf*CRT-transformed *D. discoideum* Cells

**Figure 2:**
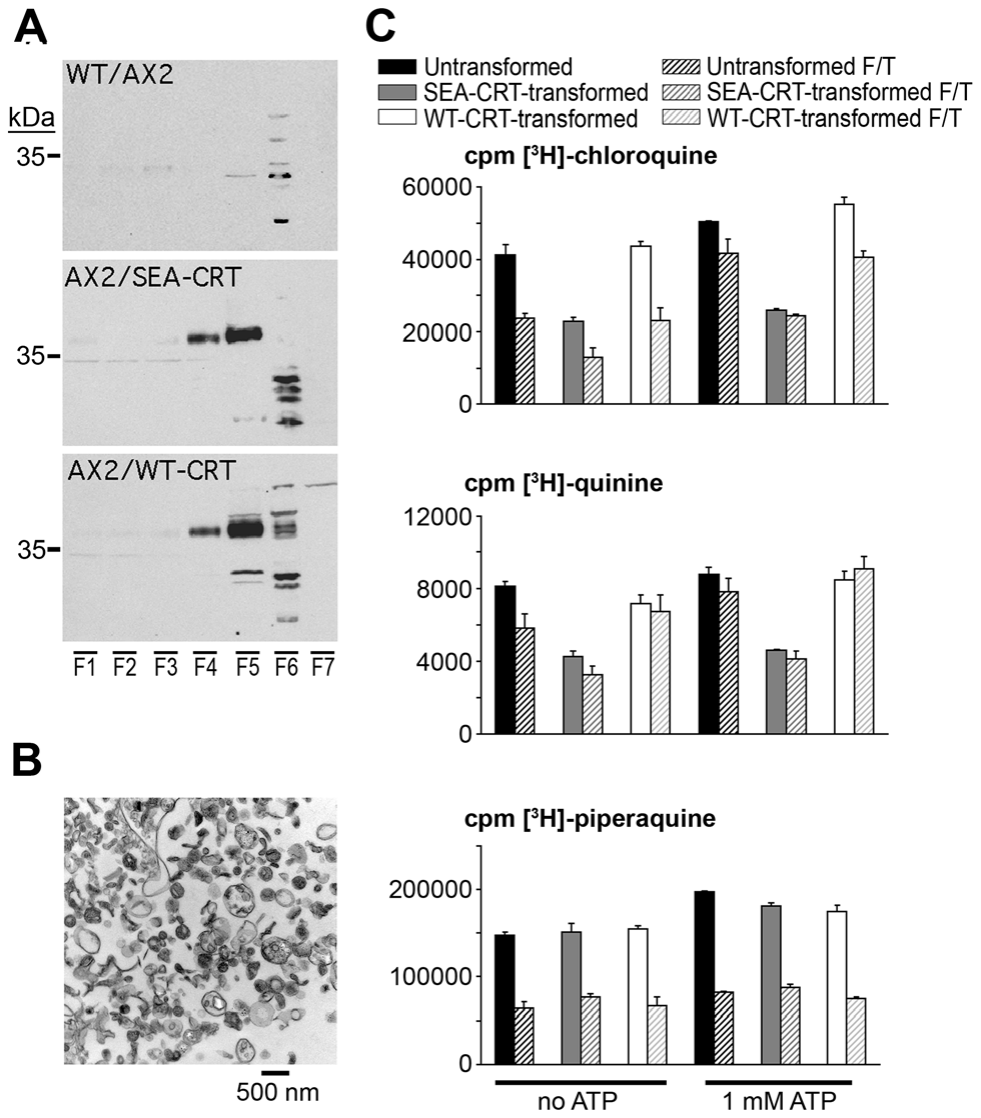
Identification of vesicles enriched for *Pf*CRT and levels of radiolabeled drug accumulation. (**A**) Semi-quantitative immunoblot (WB) analysis of membrane fractions precipitated at 1000×*g* (F2), 2000×*g* (F3), 4000×*g* (F4), 20,000×*g* (F5), and 100,000×*g* (F6). Top, middle, and bottom panels show results from the isolated vesicles of untransformed, SEA-CRT-, and WT-CRT-transformed cells. Fractions F1 and F7 represent samples of whole cells and supernatant, respectively. (**B**) Electron micrograph of a sample from F5. (**C**) Labeled drug uptake in F5 vesicles incubated for 10 min with 100 nM [^3^H]-CQ, [^3^H]-QN, or [^3^H]-PPQ in VSB with or without 1 mM ATP. Histograms compare results with fresh (solid bars) and stored frozen F/T vesicles (hashed bars) prepared from untransformed, SEA-CRT-, and WT-CRT-transformed *D. discoideum*. Error bars indicate standard deviations of measurements from three independent measurements on samples from the F/T vesicle preparation.

*D. discoideum* transformants express *Pf*CRT on the membranes of acidic vesicles that are formed downstream of the endocytosis pathway [41]. In initial attempts to isolate these vesicles, we found that concentrations of monovalent ions above 25 mM in the preparation buffer led to aggregation and alkalinization of the vesicles, which evidently swapped out their protons under these conditions. Time-consuming density gradient purification attempts with the isosmotic contrast agent iodixanol resulted in loss of vesicle acidity and loss of the CQR phenotype (from SEA-CRT-transformed vesicles), which we could not effectively restore with ATP-containing buffers or pre-incubation in acidic buffers (data not shown). We therefore developed a rapid differential centrifugation procedure in a low ion-containing, osmotically balanced sucrose buffer for isolation of the acidic vesicles. After lysis of the *D. discoideum* cells through a 5.0-μm polycarbonate filter and centrifugation of the lysate at 1000×*g* to remove nuclei, subcellular fractions were recovered from the lysates in steps of increasing centrifugation. Pellets that were recovered at 2,000×*g* and then at 4,000×*g* contained relatively small amounts of *Pf*CRT, whereas the pellet recovered in the subsequent step of centrifugation at 20,000×*g* for 10 min showed high enrichment for *Pf*CRT by semiquantitative immunoblot analysis (fraction F5; Figure 2A). Further centrifugation of the supernatant at 100,000×*g* recovered only small amounts of additional material with bands of fragments or artifact (Figure 2A; compare the bands from the untransformed cell lysate pellets with those from the SEA-CRT- and WT-CRT-transformed cell lysate pellets). The predominant segregation of *Pf*CRT with the 20,000×*g* pellet (fraction F5) and its absence from the final supernatant fraction (fraction F7) is consistent with the location of *Pf*CRT in vesicular membranes. Although fraction F4 also showed a detectable *Pf*CRT band, this signal was weaker than that of the F5 band (comparison based on equivalent pellet protein loading), and the F4 pellet carried a large amount of lysed cell debris. Experiments with fraction F4 were therefore not pursued. Signals from lower relative molecular weight bands than that of the expressed *Pf*CRT form (M*_r_* ∼48,000) were also detected in lanes F4 and F5, suggesting the presence of some polypeptides from *Pf*CRT fragments. Electron microscopy of fraction F5 confirmed a high proportion of intact vesicles (Figure 2B); this fraction was used for routine preparations and subsequent experiments. Three batches of vesicles were routinely prepared from each cell line for parallel experiments to obtain average values and standard deviations.

### Reduced Accumulation of CQ and QN but not of PPQ by SEA-CRT-transformed Vesicles

To evaluate drug accumulation by isolated vesicles, we incubated fraction F5 with [^3^H]-CQ, [^3^H]-QN or [^3^H]-PPQ in VSB with or without its component of 1 mM ATP. Vesicles from this fraction were also snap-frozen on dry ice and then thawed to test whether vesicles could be conveniently stored in sample tubes for assays at later times.

Results of these experiments showed that SEA-CRT-transformed vesicles accumulated 40–50% less [^3^H]-CQ than did corresponding samples of untransformed or WT-CRT-transformed vesicles (Figure 2C); these observations were consistent with freshly isolated and frozen/thawed (F/T) samples of vesicles. Inclusion of 1 mM ATP in the VSB generally increased the absolute counts of [^3^H]-CQ accumulation, particularly in F/T vesicles. In VSB from which ATP was omitted, the accumulation of [^3^H]-CQ in F/T vesicles was on average about 55% of the accumulation in freshly isolated vesicles from the same *D. discoideum* cell line, whereas in VSB containing 1 mM ATP, the accumulation in F/T vesicles was more than 75% of the accumulation in freshly isolated vesicles (Figure 2C).

[^3^H]-QN accumulation–although in all cases lower on an absolute scale than [^3^H]-CQ accumulation–was likewise reduced by ∼50% in SEA-CRT-transformed relative to untransformed or WT-CRT-transformed vesicles (Figure 2D). [^3^H]-QN accumulation was usually higher in freshly isolated relative to F/T vesicles, and slightly greater [^3^H]-QN accumulation was detected in presence of 1 mM ATP.

In contrast to the results with [^3^H]-CQ and [^3^H]-QN, the relative accumulation of [^3^H]-PPQ was not reduced in SEA-CRT-transformed vesicles (Figure 2E), consistent with our earlier observations on *Pf*CRT-transformed *D. discoideum* whole cells [41] and with the comparable activity of PPQ on CQ-resistant and CQ-sensitive *P. falciparum* [49]. Accumulation levels of [^3^H]-PPQ (adjusted for specific activity) were 2- to 3-fold greater in fresh relative to F/T vesicles and were increased on average ∼15% by the presence of 1 mM ATP.

### Measures of pH in Freshly Isolated and Frozen-thawed Vesicles

In previous work, Naudé *et al.* (2005) measured the signals from fluorescent dextrans in *D. discoideum* endosomes and estimated that, over the course of 1 h, vesicular pH values rose from ∼4.9–5.1 to 5.4–5.7 for post-lysosomal endosomes. These measures were in a more acidic range than previously reported values of 6.2–6.5 [50] but were nevertheless consistent with the transition of acidic lysosomal vesicles to less acidic post-lysosomal vesicles. Naudé *et al.* [41] also found that the measured increases in vesicular pH were slightly less in the WT-CRT- and SEA-CRT-transformed lines than in untransformed *D. discoideum*, with slightly more acidity observed for the vesicles of SEA-CRT relative to WT-CRT transformants. To compare pH values of vesicles from all three cell lines, we incubated intact *D. discoideum* cells with FITC coupled to dextran, which was taken up by endocytosis, and then isolated the vesicles for fluorescence measurements. After measurements of FITC fluorescence at 518 nm from excitations at 450 and 494 nm, we calculated the fluorescence ratios and estimated vesicular pH by comparison to a standard curve [42], [43]. These calculations yielded internal pHs of 6.16–6.18 for freshly isolated vesicles from untransformed, SEA-CRT-, and WT-CRT-transformed cells in VSB (Table 1; standard VSB contains 1 mM ATP), and more alkaline pH values of 6.48–6.50 in VSB without ATP. F/T vesicles exhibited pH values of 6.58–6.67 in VSB, with slightly more acidic values for the *Pf*CRT-transformed than untransformed vesicles, and pH values of 6.85–6.92 in VSB without ATP (Table 1).

**Table 1:** Estimated pH of freshly isolated and F/T vesicles isolated from untransformed and *Pf*CRT-transformed *D. discoideum*.

Preparation	Untransformed AX2*	SEA-CRT-transformed AX2	WT-CRT-transformed AX2
Fresh vesicles in VSB†	6.18±0.003	6.16±0.008	6.18±0.004
Fresh vesicles in VSB w/o ATP	6.48±0.007	6.48±0.010	6.50±0.004
F/T vesicles in VSB	6.67±0.018	6.58±0.014	6.59±0.008
F/T vesicles in VSB w/o ATP	6.92±0.078	6.85±0.035	6.86±0.050

* D. discoideum AX2 line.

† Standard VSB contains 1 mM ATP.

The lower accumulation of [^3^H]-CQ in more alkaline F/T vs. freshly isolated vesicles (Fig. 2C) is in line with the behavior of CQ as a di-protic weak base in compartments of different pH. Note, however, that the fractional reduction of [^3^H]-CQ accumulation in SEA-CRT- vs. WT-CRT-transformed or untransformed vesicles was similar in the freshly isolated and F/T preparations (Figure 2C). In addition, based on our average of pH 6.49 for fresh vesicles and pH 6.88 for F/T vesicles, the theory of di-protic weak base accumulation would predict an 83% reduction in accumulation, much more than observed and again suggesting a major influence of a factor other than pH in CQ accumulation.

Considering these observations and the advantage and efficiency of F/T vesicle preparations for experimental studies, we performed all further work on F/T vesicles, thus providing consistent and reproducible conditions for comparisons and evaluations.

### Substantial reversal of the CQR Phenotype in Isolated Vesicles by OS or MCB but only limited reversal by VP

**Figure 3:**
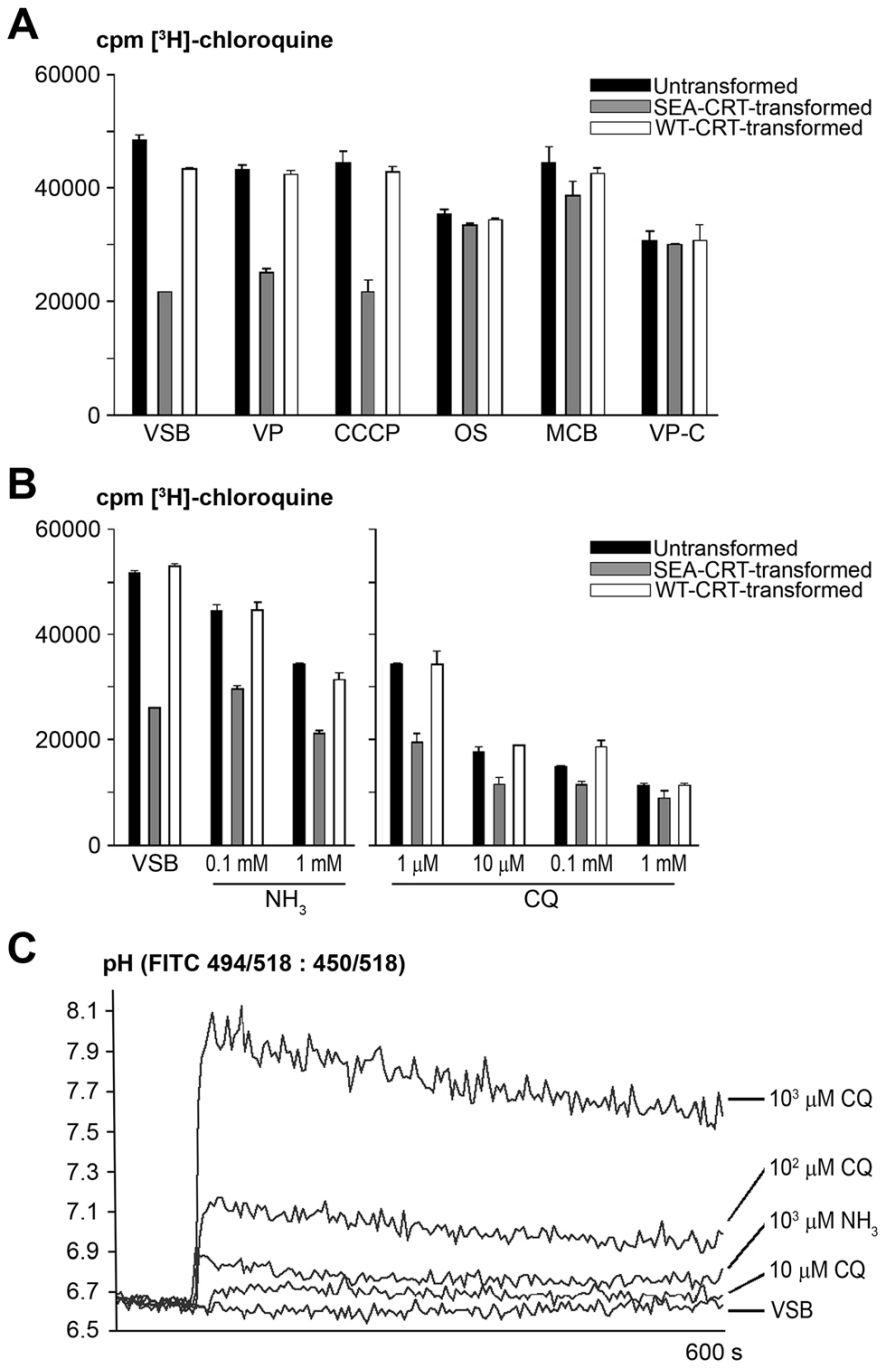
Effects of buffer conditions on [^3^H]-CQ accumulation and pH in F/T vesicles from untransformed, SEA-CRT-, and WT-CRT-transformed *Dictyostelium discoideum.* (**A**) [^3^H]-CQ uptake by F/T vesicles in VSB vs. vesicles exposed to 80 μM VP in VSB, 2 μM CCCP in VSB, OS, or MCB. Levels in vesicles isolated from whole cells pre-incubated in 100 nM [^3^H]-CQ and 80 μM VP are shown at the right (VP-C). (**B**) Radiolabel uptake by F/T vesicles exposed to 100 nM [^3^H]-CQ in VSB containing various concentrations of NH_3_ (0.0, 0.1 or 1 mM; left panel) or various concentrations of additional unlabeled CQ (1, 10, 100 or 1000 µM; right panel). (**C**) pH determinations of isolated FITC-dextran loaded vesicles incubated for 10 min in the presence of various concentrations of CQ or 1 mM ammonia. Error bars indicate standard deviations of measurements from three independent measurements on samples from the F/T vesicle preparation.

In agreement with the CQR phenotype of intact SEA-CRT-transformed *D. discoideum* cells (Figure 1B), isolated SEA-CRT-transformed vesicles accumulated 40 – 45% less [^3^H]-CQ than untransformed or WT-CRT-transformed vesicles in the absence or presence of 1 mM ATP (Figures 2C and 3A). Inclusion of VP, however, had only a limited effect on [^3^H]-CQ accumulation in isolated vesicles relative to its effect on [^3^H]-CQ accumulation in whole cells: SEA-CRT-transformed vesicles in the presence of 80 µM VP exhibited just ∼25% of the reversal effect that was observed with SEA-CRT-transformed whole cells in 80 µM VP (compare Figures 3A and 1B). CCCP did not reverse the CQR phenotype in isolated vesicles (Figure 3A), consistent with the fact that the action of CCCP is by the inhibition of mitochondrial ATP production in whole cells (Figure 1B, C).

Although VP provided only limited reversal of the reduced CQR accumulation phenotype of isolated SEA-CRT-transformed vesicles, subjection of these vesicles to 10 min in OS (VSB containing 100 mM sucrose instead of 210 mM sucrose) followed by addition of an equal volume of VSB containing 320 mM sucrose (achieving a final sucrose concentration of 210 mM) increased their [^3^H]-CQ accumulation to the levels of similarly-treated untransformed and WT-CRT-transformed vesicles (OS, Figure 3A). Substituting MCB for VSB likewise resulted in complete reversal of the CQR phenotype (Figure 3A).

Failure of VP to equalize [^3^H]-CQ accumulation in isolated transformed vesicles as it does in intact *D. discoideum* cells suggested that full VP reversal of the CQ resistance phenotype depends on a vesicle property that is altered during the isolation process. To explore this possibility, we isolated the vesicles from cells that had been pre-incubated with [^3^H]-CQ in the presence of 80 µM VP. Results showed that such isolated vesicles from SEA-CRT-, WT-CRT-transformed, and untransformed cells contained equivalent amounts of [^3^H]-CQ (Figure 3A; VP-C). Addition of cellular lysate supernatants to isolated vesicle suspensions prior to their exposure to [^3^H]-CQ and VP did not improve their VP-reversal phenotype (data not shown).

### Effect of VP, CCCP, OS, and MCB on the pH of F/T vesicles

Although VP and CCCP effectively eliminated the proton gradients across membranes of acidic vesicles in intact cells (Figure 1, D–F), these agents produced relatively small effects on isolated F/T vesicles in VSB, raising their pH to 6.73–6.84 from 6.58–6.67 (compare entries in Tables 1 and 2). In contrast, F/T vesicles subjected to OS for 10 min yielded pH values of 7.47–7.54 (Table 2), about 0.2 pH unit more alkaline than the pH of OS itself. The explanation for these high pH values is not clear. One possibility is that restoration of vesicle ion concentrations after osmotic shock involves exit of protons and consequent alkalinization through cation/H^+^ exchange systems. However these high pH values might be explained, note that relative levels of CQ accumulation in the alkalinized vesicles after osmotic shock were not greatly different from those of F/T vesicles in VSB (except for reversal of the reduced drug accumulation phenotype in SEA-CRT-transformed vesicles) or from those of vesicles isolated from *D. discoideum* cells pre-exposed to [^3^H]-CQ in the presence of 80 µM VP (Figure 3A).

**Table 2:** Effect of VP, CCCP, OS, and high-sucrose MCB on pH of F/T vesicles isolated from untransformed and PfCRT-transformed *D. discoideum*.

Preparation	Untransformed AX2*	SEA-CRT-transformed AX2	WT-CRT-transformed AX2
VSB† +100 µM VP	6.82±0.082	6.84±0.069	6.77±0.025
VSB +2 µM CCCP	6.83±0.049	6.73±0.036	6.83±0.032
OS	7.54±0.040	7.47±0.051	7.51±0.005
MCB (240 mM sucrose)†	6.80±0.180	6.93±0.011	6.76±0.023

* D. discoideum AX2 line.

† Standard VSB and MCB contain 1 mM ATP.

In contrast to vesicles exposed to OS, vesicles in a modified buffer in which monovalent ions were replaced with an osmotically equivalent amount of sucrose (MCB) remained slightly acidic at pH values of 6.76–6.93 (Table 2). Despite the difference between these pH values and the alkaline pH values of osmotically shocked vesicles, [^3^H]-CQ accumulation in the vesicles did not greatly differ (Figure 3A), again indicating an important role for a factor other than pH in the CQR phenotype.

### Effects of Ammonia and Unlabeled CQ on [^3^H]-CQ Accumulation and Vesicle pH

As an additional evaluation of [^3^H]-CQ accumulation after treatments that alter vesicular pH, we exposed isolated F/T vesicles to different concentrations of ammonia in VSB (Figure 3B, left panel). In the presence of 0.1 mM ammonia, the CQR phenotype of SEA-CRT-transformed vesicles was blunted from 50% to 35% reduced relative accumulation by the opposite effects of an increase of [^3^H]-CQ in SEA-CRT-transformed vesicles and a relative decrease of [^3^H]-CQ in untransformed and WT-CRT-transformed vesicles. Increasing the ammonia concentration to 1 mM achieved further blunting of the CQR phenotype but it did not equalize the [^3^H]-CQ levels of SEA-CRT-transformed vesicles to those of untransformed or WT-CRT-transformed vesicles (Figure 3B, left panel). These results were in contrast to the full equalization achieved with 100 µM ammonia in the different lines of whole cells (Figure 1) but were consistent with the observation that VP likewise had a limited effect on the CQR phenotype of isolated vesicles (Figure 3A).

We also studied the effects of unlabeled (cold) CQ on [^3^H]-CQ accumulation by isolated vesicles. In these experiments (Figure 3B, right panel), [^3^H]-CQ was included in the suspension buffer with various concentrations of unlabeled CQ. Results from these experiments showed that unlabeled CQ blunted the CQR phenotype with greater potency than ammonia: 1 µM unlabeled CQ reduced [^3^H]-CQ accumulation about as much as did 1 mM ammonia. Higher concentrations of unlabeled CQ differentially reduced [^3^H]-CQ accumulation in SEA-CRT-transformed relative to untransformed and WT-CRT-transformed vesicles and, at 1 mM, caused similar levels of accumulation (Figure 3B, right panel) due to a ∼65% reduction of [^3^H]-CQ accumulation in SEA-CRT-transformed vesicles vs. ∼80% reductions in untransformed and WT-CRT-transformed vesicles. In contrast to its effect on whole cells (compare Figure 1A), unlabeled CQ did not increase the accumulation of [^3^H]-CQ in vesicles from SEA-CRT-transformed cells at concentrations of 10 and 100 μM.

To estimate the pH values of vesicles under these experimental conditions, we obtained continuous measures of fluorescence ratios from F/T FITC-loaded vesicles in VSB as described for Table 1. FITC-loaded vesicles from untransformed cells showed little change in these ratios, indicating an average pH 6.6–6.7 over the 10-min measurement period (Figure 3C). However, significant alkalinization was observed when the vesicles were exposed to ammonia or unlabeled CQ. A 1 mM concentration of ammonia immediately raised the vesicular pH to 6.9, which settled to 6.8 after 10 min (little change of pH occurred with 0.1 mM ammonia; data not shown). In comparison to ammonia, added concentrations of CQ affected vesicular pH more powerfully. For example, 0.1 mM CQ produced a rise from initial pH 6.6–6.7 to pH 7.1–7.2, which settled to pH 7.0–7.1 after 10 min. A much greater effect was observed after the addition of 1 mM CQ: endosomal pH quickly rose to pH 7.9–8.1 before decreasing over 10 min to pH 7.6–7.7 (Figure 3C).

Although 1 mM ammonia raised vesicular pH to a more alkaline value than 10 µM CQ (Figure 3C), we note that [^3^H]-CQ accumulation was 50–100% greater in the presence of 1 mM ammonia than in the presence of 10 µM unlabeled CQ (Figure 3B). This was opposite of what would be expected if pH alone were the major determinant of CQ accumulation. Moreover, the accumulation of [^3^H]-CQ did not differ greatly in the presence of 10 µM, 0.1 mM or 1 mM unlabeled CQ, despite the large differences in the pH of vesicles exposed to these CQ concentrations (compare Figure 3, B and C).

### Effects of Ionophores on [^3^H]-CQ Accumulation by Whole Cells and Isolated Vesicles

**Figure 4:**
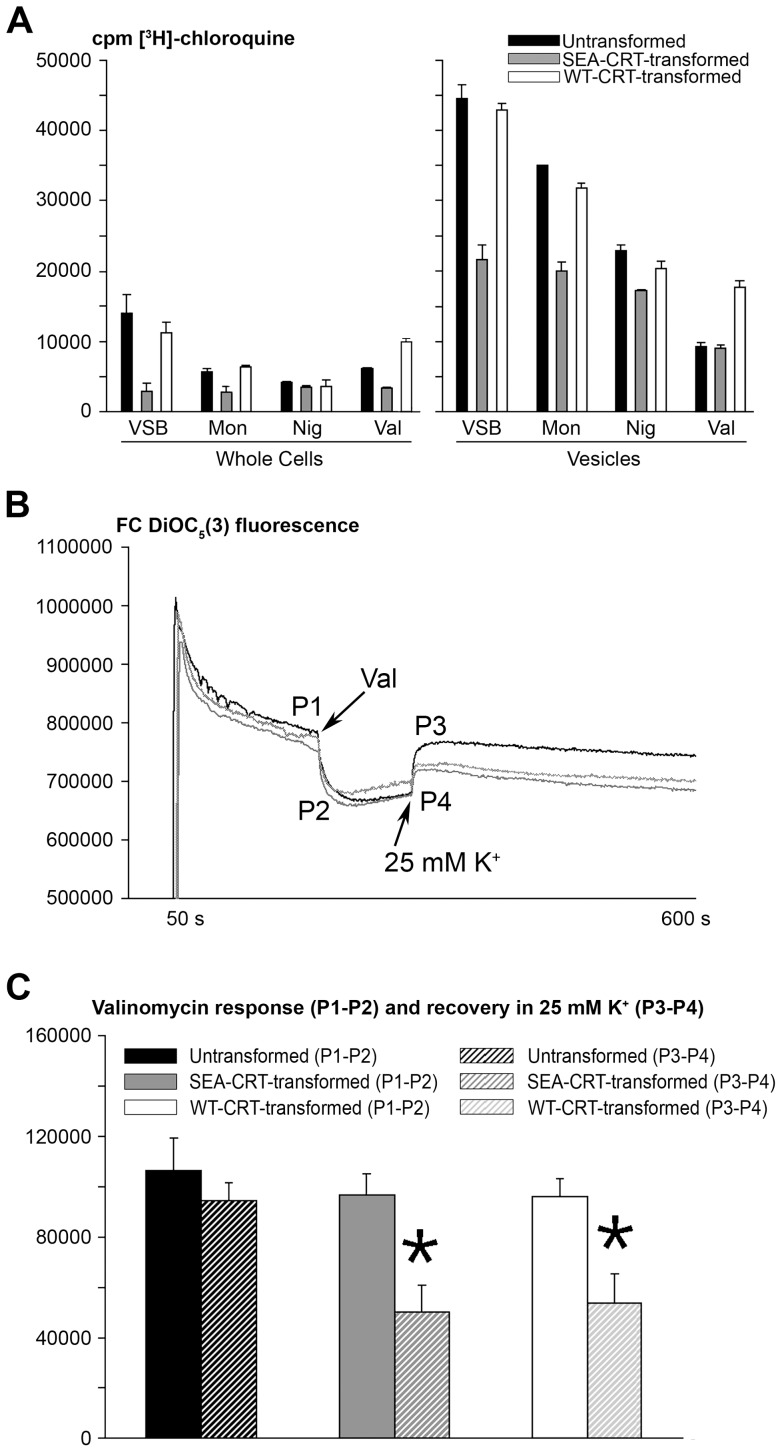
Influence of ionophores on [^3^H]-CQ accumulation by whole cells and isolated vesicles from untransformed and *Pf*CRT-transformed *Dictyostelium discoideum*. (**A**) Labeled drug uptake by whole cells (in PB) or vesicles (in VSB) in the presence of 100 nM [^3^H]-CQ and 2 μM concentrations of monensin (Mon), nigericin (Nig), or valinomycin (Val). Cells were incubated in the buffers for 10 min before determination of [^3^H]-CQ uptake. Note the similar accumulations of [^3^H]-CQ by untransformed and SEA-CRT-transformed vesicles in the presence of Val. Error bars indicate standard deviations of measurements from three independent measurements. (**B**) Representative DiOC_5_(3) fluorescence traces indicating membrane potential changes in untransformed (black), SEA-CRT-transformed (grey), and WT-CRT-transformed (light grey) F/T vesicles in MCB. Val (2 μM) was added at 200 s, and K^+^ (25 mM) was added at 300 s. P1, P2, P3, and P4 indicate points where fluorescence intensity measurements were taken from the tracings to determine the magnitude of hyperpolarization by valinomycin (P1, fluorescence level before valinomycin is added; P2, trough fluorescence) and the magnitude of depolarization after addition of 25 mM K^+^ (P3, peak fluorescence after K+ addition; P4, fluorescence level before K^+^ is added). FC, fluorescence counts. (**C**) Average valinomycin response (P1–P2) and recoveries of DiOC_5_ (3) fluorescence (P3–P4) before and after addition of 25 mM K^+^ in eight independent experiments. Asterisks (*) indicate significant difference from the average result with untransformed vesicles (P<0.005; n = 8).

Figure 4A compares [^3^H]-CQ accumulation results from tests of various ionophores on intact *D. discoideum* cells in PB and on F/T vesicle preparations in VSB. Monensin is an electroneutral Na^+^/H^+^-antiporter with little or no direct effect on membrane potential [51]. In intact untransformed and WT-CRT-transformed *D. discoideum* cells, monensin reduced CQ accumulation by approximately 50%, whereas this drug showed no detectable effect on [^3^H]-CQ accumulation in SEA-CRT-transformed cells (Figure 4A, left panel). Likewise, monensin treatment reduced [^3^H]-CQ accumulation more in untransformed and WT-CRT-transformed F/T vesicles than in SEA-CRT-transformed vesicles (Figure 4A, right panel). Consistent with the moderate reduction of [^3^H]-CQ accumulation in untransformed and WT-CRT-transformed vesicles, monensin produced a moderate effect on vesicular pH only (Table 3).

**Table 3:** Effects of ionophores on pH of F/T vesicles isolated from untransformed and *Pf*CRT-transformed *D. discoideum*.

Preparation	Untransformed AX2*	SEA-CRT-transformed AX2	WT-CRT-transformed AX2
VSB† +2 µM Monensin	6.84±0.041	6.93±0.027	6.82±0.027
VSB +2 µM Nigericin	7.27±0.029	7.27±0.082	7.24±0.054
MCB† +2 µM Valinomycin +25 mM K^+^	6.90±0.012	6.52±0.048	6.54±0.094

* D. discoideum AX2 line.

† Standard VSB and MCB contain 1 mM ATP.

Nigericin, an electroneutral K^+^/H^+^-antiporter that also has little or no direct effect on membrane potential [51], produced greater reductions than monensin on [^3^H]-CQ accumulation by untransformed and WT-CRT-transformed cells (Figure 4A, left panel). Nigericin likewise produced greater reductions than monensin of [^3^H]-CQ accumulation in the isolated F/T vesicles from transformed and untransformed cells (Figure 4A, right panel), and it had a greater effect on vesicular pH, producing a slightly alkaline condition (Table 3). In comparisons of [^3^H]-CQ accumulation by SEA-transformed *vs*. untransformed or WT-CRT-transformed vesicles, only ∼15–25% differences were found for nigericin-treated vesicles whereas ∼35–40% and ∼45–50% differences were found for monensin-treated and untreated vesicles, respectively (Figure 4A, right panel).

Valinomycin, a K^+^ ionophore that can produce strong effects on membrane potential also caused marked reductions of [^3^H]-CQ accumulation in untransformed *D. discoideum* cells and isolated vesicles; however, in contrast to the results with monensin and nigericin, valinomycin reduced [^3^H]-CQ accumulation in untransformed cells to a much greater extent than in WT-CRT or SEA-CRT-transformed cells (Figure 4A, left panel). In isolated F/T vesicles, valinomycin produced the greatest reductions of all the ionophores, to low levels of [^3^H]-CQ accumulation that were similar in untransformed and SEA-CRT transformed vesicles, and to a level in WT-CRT-transformed vesicles that was less than the level seen in the corresponding nigericin-treated vesicles (Figure 4A, right panel). Of particular note, a much greater effect of valinomycin was evident on untransformed F/T vesicles relative to F/T vesicles transformed with either WT-CRT or SEA-CRT. Valinomycin moreover produced a change in the pH of untransformed vesicles that was opposite to the effect on vesicles from *Pf*CRT-transformed cells, *i.e.* exposure of valinomycin to the *Pf*CRT-transformed vesicles caused an acid shift to pH 6.53–6.54 whereas the valinomycin exposure to untransformed vesicles shifted the pH upward to 6.9 (Table 3).

### Effects of Valinomycin and K^+^ on Vesicle Membrane Potential

In light of the data indicating an important role of vesicle membrane potential in the CQR phenotype [26], [27], [30], [36], [37], we designed experiments to test for different membrane potential responses of *Pf*CRT-transformed *vs.* untransformed vesicles. Membrane potential changes can be detected by real-time traces of the fluorescent potential probe DiOC_5_(3) which accumulates and forms voltage-dependent complexes in membranes, a process that quenches the fluorescence of the monomers [52].

Isolated F/T vesicles suspended in VSB showed little or no detectable change in DiOC_5_(3) traces after addition of 2 μM valinomycin (data not shown); however, vesicles suspended in K^+^-free MCB all showed a marked enhancement of fluorescence quenching after addition of 2 μM valinomycin (Figure 4B), indicating increased negative membrane potential from the valinomycin-induced efflux of vesicular K^+^ ions out of F/T vesicles. Subsequent addition of 25 mM K^+^ to valinomycin-treated vesicles in MCB reversed the additional component of DiOC_5_(3) quenching by removing the negative electrochemical contribution of the original inside-out K^+^ gradient across the valinomycin-treated membrane (Figure 4B). Whereas this reversal of quenching was complete with untransformed vesicles, it was not complete with transformed either WT-CRT- or SEA-CRT-transformed vesicles. These differences of reversal were quantified from each trace by measurements of the fluorescence levels after (Figure 4B, P3) and before (Figure 4B, P4) the addition of 25 mM K^+^. In results from eight separate experiments, the WT-CRT- and SEA-CRT-transformed vesicles showed ∼ 55% of the K^+^ step response seen with untransformed vesicles in the presence of valinomycin (Figure 4C). No significant differences in the additional polarization from valinomycin alone were detected among the traces of untransformed, SEA-CRT-, or WT-CRT-transformed vesicles (Figure 4C, P1–P2).

## Discussion

In previous work, Naudé *et al*. [41] showed that *D. discoideum* cells acquired the CQR phenotype of reduced CQ accumulation when these cells were transformed with the mutant SEA but not the WT form of *Pf*CRT. Changes of intravesicular pH did not account for these findings, implicating another factor in the mechanism of reduced CQ accumulation by mutant *Pf*CRT. Moreover, this mechanism demonstrated structural specificity, as reduction of CQ accumulation in *D. discoideum* transformants did not extend to PPQ, a bisquinoline analog of CQ that is effective against CQ-resistant parasites. Our present data confirm and extend these observations of structural specificity of the *Pf*CRT-mediated mechanism and the involvement of a factor other than pH on CQ accumulation by whole *D. discoideum* cells. The results indicate that CQ accumulation in *Pf*CRT-transformed vesicles from *D. discoideum* cells is influenced by vesicle membrane potential. These findings are consistent with the conclusions of previous reports that: (1) the *Pf*CRT-mediated CQR phenotype of *P. falciparum* depends upon DV membrane potential [26], [27], [30], [36], [37]; and (2) *Pf*CRT, in its CQR form, operates as a passive carrier under the influence of membrane potential or, less likely, as a voltage-dependent channel through which protonated CQ can pass, allowing the diffusion of protonated CQ down its electrochemical gradient and out of the DV [24]–[31].

Treatment of *D. discoideum* cells with VP or CCCP neutralized the acid compartments of their vesicles while producing levels of [^3^H]-CQ accumulation above the level observed in untreated, SEA-CRT-transformed cells (Figure 1B, D–F); [^3^H]-CQ accumulation in the VP- or CCCP-treated SEA-CRT-transformed cells also showed little or no difference from the accumulation in identically treated untransformed or WT-CRT-transformed cells. pH dependence of the CQR phenotype is consistent with results from *Pf*CRT-transformed *X. laevis* oocytes, in which the reduced accumulation phenotype conferred by *Pf*CRT^CQR^ relative to *Pf*CRT^CQS^ was lost at pH ∼7.4 and above [30]. It also explains reversal of the CQR phenotype by accumulated levels of NH3 or CQ as protonated weak bases (Figure 1A and 3B, C). The reason for this overall increase of [^3^H]-CQ accumulation in VP- and CCCP-treated cells remains to be established (either transformed or untransformed; Figure 1B). A possible explanation is that endogenous transporters of the *D. discoideum* vesicles can act on protonated CQ with different efficiencies at acidic or neutral pH.

Isolated vesicles showed only limited reversal of CQR phenotype in 80 μM VP even though the SEA-CRT-transformed *D. discoideum* cells showed nearly complete reversal at this same concentration of VP (compare Figures 3A and 1B). This limited reversal likely relates to the effect of pH on the ability of VP, a weak base, to accumulate and inhibit CQ efflux. As vesicles mature in the *D. discoideum* endosomal pathway, they change over the course of an hour from an acidic lysosomal to a less acidic post-lysosomal condition [53]. Presence of post-lysosomal vesicles in our preparations would be consistent with the relatively high pH of fresh isolated vesicles (pH ∼6.5 without ATP) and the limited ability of ATP in Mg^++^-containing buffer to lower the pH of these vesicles to more acidic levels (Table 1). VP presumably would not have been able to accumulate in isolated SEA-CRT-transformed vesicles to the levels necessary for full reversal of the reduced CQ accumulation phenotype. This explanation is consistent with the facts that VP reversal is firmly linked to *Pf*CRT mutations in *P. falciparum* [54], [55] and that VP specifically inhibits the transport of CQ by mutant forms of *Pf*CRT expressed at the surface of *X. laevis* oocytes [30].

CCCP fully reversed the CQR phenotype of intact SEA-CRT-transformed *D. discoideum* cells but showed no such effect on isolated vesicle preparations (compare Figure 1B and Figure 3A). Notably, the reversal of the CQR phenotype of intact cells by CCCP was achieved at a concentration (2 µM) that was about 50-fold less than that required for complete reversal by VP or NH_3_ (80–100 µM). This large difference in concentration is explained by the CCCP's mechanism of action which inhibits mitochondrial ATP production and thereby deprives the intracellular vesicles of the energy they require for acidification (Figure 1B–F). Thus, neutralization of the intracellular vesicles by CCCP was thus achieved by a mechanism different from weak base lysosomotropic action. Isolated vesicles treated with CCCP showed small if any change in CQ levels, indicating little influence of this protonophore on their accumulation phenotype (Figure 3A).

In contrast to the limited effects of VP and CCCP on isolated vesicles, exposure of isolated vesicles to OS or to MCB with the aim to perturb proton and other ion gradients across the vesicular membrane successfully reversed the CQR phenotype (Figure 3A). The intravesicular pH levels after these exposures were less acidic in MCB (pH ∼6.8–6.9, slightly higher than pH ∼6.6–6.7 of F/T vesicles in VSB) and alkalinized in OS (pH ∼7.5) (Table 2). The finding that MCB exposure reversed the CQR phenotype without a large pH shift provides further evidence that conditions affecting ion distributions and membrane potential are important determinants of CQ accumulation and *Pf*CRT activity in the vesicles.

To further explore the effects of ion distributions and membrane potential in vesicles, we evaluated [^3^H]-CQ accumulation, vesicle pH and DiOC5(3) fluorescence in the presence of the ionophores monensin, nigericin, and valinomycin. The electroneutral transporters monensin (Na^+^/H^+^) and nigericin (K^+^/H^+^) raised vesicle pH levels to ∼6.8–6.9 and ∼7.2–7.3, respectively (Table 3). These treatments also caused marked reductions of [^3^H]-CQ accumulation in untransformed or WT-CRT-transformed vesicles relative to comparatively minor reductions of [^3^H]-CQ accumulation in SEA-CRT-transformed vesicles (Figure 4A). In whole cells, the reductions of [^3^H]-CQ accumulation by monensin and nigericin were opposite to the effects of VP and CCCP which increased [^3^H]-CQ levels despite neutralization of the vesicles (Figure 1B, D–F); these findings reinforce the case for the roles of electrochemical gradients and additional endogenous transporters on [^3^H]-CQ accumulation. Compared to monensin-treated vesicles, nigericin-treated vesicles showed smaller difference between the [^3^H]-CQ accumulation of WT-CRT-transformed *vs.* SEA-CRT-transformed or untransformed vesicles (Figure 4A). As noted above for vesicles alkalinized by other means, these smaller differences of [^3^H]-CQ accumulation in nigericin-treated vesicles at pH ∼7.2–7.3 is consistent with the results from *Pf*CRT-transformed *Xenopus* oocytes, in which the reduced accumulation phenotype conferred by *Pf*CRT^CQR^ relative to *Pf*CRT^CQS^ was not evident at pH ∼7.4 and above [30].

In experiments with the K^+^ uniporter valinomycin, *Pf*CRT in both its native WT-CRT and mutant SEA-CRT forms blunted the ability of valinomycin to reduce [^3^H]-CQ accumulation in transformed relative to untransformed vesicles (Figure 4A). The near neutralization of untransformed vesicles (pH ∼6.9; Table 3) but not of *Pf*CRT-transformed vesicles (pH ∼6.5) in MCB +25 mM K^+^ may be explained by a *Pf*CRT action that alters the expression of endogenous channels or transporters involved in the maintenance of pH and membrane potential. An alternative explanation for these results could be that the effect of inward K^+^ uniport through valinomycin is blunted in the transformed vesicles by outward, *Pf*CRT-mediated movement of K^+^ in direct or indirect exchange for protons, but for reasons discussed below this possibility is unlikely. Different pH values between untransformed and *Pf*CRT-transformed vesicles were not observed after their alkalinization by nigericin treatment, presumably because the large, electroneutral K^+^_(inward)_/H^+^_(outward)_ exchange from nigericin was not affected by any activity of *Pf*CRT.

Our findings from ionophore treatment, together with the already existing evidence for effects of membrane potential on *Pf*CRT-mediated [^3^H]-CQ accumulation [26], [27], [30], [36], [37], led us to explore the influence of valinomycin and K^+^ on DiOC5(3) fluorescence from untransformed and *Pf*CRT-transformed vesicles. DiOC5(3) fluorescence quenching was rapid and indistinguishable between the untransformed and *Pf*CRT-transformed vesicles suspended in MCB (no K^+^), indicative of an inside negative membrane potential not altered by the presence of *Pf*CRT (Figure 4B). Treatment of these vesicles with valinomycin exhibited increased membrane polarization (hyperpolarization) because of valinomycin's contribution of an additional outward-directed flux of intravesicular K^+^. This hyperpolarization could be completely reversed by external K^+^ (25 mM) only for untransformed vesicles; little more than half of the hyperpolarization of valinomycin-treated WT-CRT- or SEA-CRT-transformed vesicles could be reversed by application of external K^+^ (Figure 4B, C). In experiments with *Xenopus* oocytes, Martin *et al.* [30] found that the membrane potential of *Pf*CRT-transformed oocytes was significantly less responsive than that of non-injected oocytes to the replacement of extracellular Na^+^ by K^+^, suggesting that decreased expression of endogenous channels or transporters ensured the maintenance of membrane potential in the presence of *Pf*CRT. Activation of endogenous transporters in *Xenopus* oocytes expressing *Pf*CRT has also been reported [40]. It is possible that the effect of *Pf*CRT on transformed *D. discoideum* vesicles was similarly offset by altered expression of their endogenous membrane proteins, resulting in no overall change of membrane potential and reduced ability of external K^+^ to reverse the effect of valinomycin treatment. A less likely possibility for this reduced ability of K^+^ to reverse the valinomycin-induced hyperpolarization of *Pf*CRT-transformed vesicles might be that *Pf*CRT transports K^+^ in symport or antiport with other substrates. However, we have been unable to identify a precedent for such action on K^+^ in the DMT superfamily of proteins that share evolutionary affinity with *Pf*CRT and its orthologs [12], [13]; known K^+^ transporters and channels reside in families other than those of the DMT superfamily [56] (http://www.tcdb.org/).

Although genetic studies have established that mutations of *Pf*CRT are the central determinant of CQR in *P. falciparum*, an understanding of the native role of *Pf*CRT has remained elusive and the biophysical processes involved in resistance are yet to be clarified. Further investigations of expressed *Pf*CRT in vesicles and model membrane systems should enable advances on several key questions, among them: how electrochemical gradients and membrane potential are linked to *PfCRT*-mediated reductions of drug accumulation in the CQR phenotype; how VP interacts with mutant *Pf*CRT to reverse the CQR phenotype; and structure-function relationships of *Pf*CRT including a molecular description of CQ binding in the mechanism of drug resistance.
